# Rational incorporation of defects within metal–organic frameworks generates highly active electrocatalytic sites†

**DOI:** 10.1039/d1sc00573a

**Published:** 2021-04-29

**Authors:** Nina Heidary, Daniel Chartrand, Amandine Guiet, Nikolay Kornienko

**Affiliations:** Department of Chemistry, Université de Montréal 1375 Avenue Thérèse-Lavoie-Roux Montréal QC H2V 0B3 Canada nikolay.kornienko@umontreal.ca; Institut des Molécules et Matériaux du Mans (IMMM), UMR 6283 CNRS, Le Mans Université Avenue Olivier Messiaen 72085 Le Mans France

## Abstract

The allure of metal–organic frameworks (MOFs) in heterogeneous electrocatalysis is that catalytically active sites may be designed *a priori* with an unparalleled degree of control. An emerging strategy to generate coordinatively-unsaturated active sites is through the use of organic linkers that lack a functional group that would usually bind with the metal nodes. To execute this strategy, we synthesize a model MOF, Ni-MOF-74 and incorporate a fraction of 2-hydroxyterephthalic acid in place of 2,5-dihydroxyterephthalic acid. The defective MOF, Ni-MOF-74D, is evaluated *vs.* the nominally defect-free Ni-MOF-74 with a host of *ex situ* and *in situ* spectroscopic and electroanalytical techniques, using the oxidation of hydroxymethylfurtural (HMF) as a model reaction. The data indicates that Ni-MOF-74D features a set of 4-coordinate Ni–O_4_ sites that exhibit unique vibrational signatures, redox potentials, binding motifs to HMF, and consequently superior electrocatalytic activity relative to the original Ni-MOF-74 MOF, being able to convert HMF to the desired 2,5-furandicarboxylic acid at 95% yield and 80% faradaic efficiency. Furthermore, having such rationally well-defined catalytic sites coupled with *in situ* Raman and infrared spectroelectrochemical measurements enabled the deduction of the reaction mechanism in which co-adsorbed *OH functions as a proton acceptor in the alcohol oxidation step and carries implications for catalyst design for heterogeneous electrosynthetic reactions en route to the electrification of the chemical industry.

## Abstract 2

The allure of metal–organic frameworks (MOFs) in heterogeneous electrocatalysis is that catalytically active sites may be designed *a priori* with an unparalleled degree of control.

## Graphical abstract

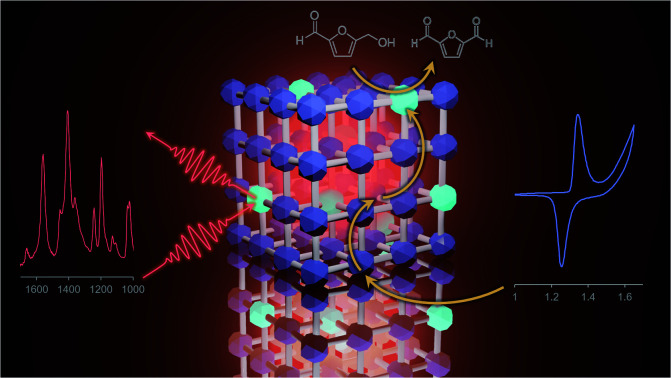

## Introduction

The utilization of metal and covalent organic frameworks (MOFs and COFs) in electrochemical systems is increasingly attracting interest because of the unique capabilities of these structures.^1–5^ Through judicious selection of the organic linker and metal nodes, the porosity, internal chemistry, conductivity, active site physical and electronic structure can all be chosen *a priori* to generate the ideal catalyst. This degree of control is unmatched by conventional heterogeneous and molecular materials, thus opening up unique parameter spaces to explore.

In regard to the tuning of the active site several strategies have been explored in recent years. Molecular catalysts serving as the organic linker within MOFs and COFs gave rise to several highly functional catalytic systems for the hydrogen evolution reaction (HER),^6,7^ CO_2_ reduction reaction (CO_2_R),^8–12^ and oxygen reduction reaction (ORR).^13^ The activity of the molecular active could be further tuned through electron withdrawing functional groups grafted within the COF structures.^14^ The metal nodes of MOFs could also serve as active sites for the HER^15^ and the oxygen evolution reaction (OER),^16–19^ and the activity these can be augmented through lattice strain^20^ or modulation of the linker electronic structure. Finally, the reaction environment within MOFs and related systems can be tuned to impart secondary coordination sphere effects in an enzyme-inspired fashion.^21^ This has recently been exemplified through the grafting of pyridine units that stabilize the CO_2_˙^−^ intermediate on a Co-protoporphyrin active site en route to the reduction of CO_2_ to CO in a metal–organic layer.^22^

One strategy recently established in the MOF community is the use of organic linkers that are missing functional groups that bind to the metal nodes. Successful incorporation of such missing functional groups consequently results in a fraction of the metal nodes that are coordinatively unsaturated. The resultant defect sites have primarily been applied in gas sorption,^23^ chemical separations,^24^ liquid phase catalytic transformations^25^ and proton conductivity.^26^

In the context of electrocatalysis, these unsaturated sites have the potential to be highly active and their rational design and incorporation within an electrocatalytic MOF stands to augment its activity. This has recently been demonstrated for the OER,^27^ in which unsaturated Co nodes exhibited a favorable electronic structure to facilitate water oxidation. As this strategy is rather nascent, neither a complete understanding of the resultant undercoordinated sites nor the methodology to evaluated them is yet established. We envisioned that pushing the envelope of analytical methodology is thus necessary to make substantial headway in this direction. Against this backdrop, we moved to explore a missing hydroxyl linker MOF with a unique combination of *in situ* Raman and infrared spectroscopies and electrochemical techniques using the aqueous electrooxidation of hydroxymethylfurfural (HMF)^28^ as a model reaction. This reaction represents an important challenge in the emerging area of electrochemical biomass valorization, which carries the potential to use renewable electricity to generate, from abundant raw ingredients, many of the fuels and chemicals that power society.^29^ While HMF oxidation is beginning to be investigated using principally metal oxide catalysts, there is much room for improvement in terms of minimizing overpotential and understanding the surface reaction mechanism.^29,30^ Emerging from our experiments is the deduction of a unique dual-site catalytic mechanism, carrying implications towards the design of next-generation electrosynthetic systems executing a wide gamut of catalytic transformations.

**Fig. 1:**
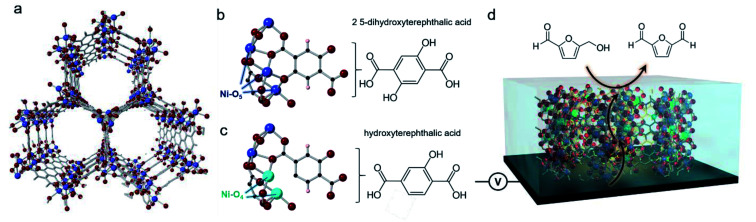
Ni-MOF 74 contains cylinder shaped pores and chains of 5-coordinate Ni oxide units (a). The 2,5-dihydroxyterepthalic acid organic linker, will bind through both the carboxylate and alcohol units (b) and replacing it with 2-hydroxyterepthalic acid will result in defect sites of 4-coordinate Ni sites (c). We studied the effects of these defect sites with electrochemical and spectroscopic techniques, using the oxidation of HMF as a model reaction (d).

We chose Ni-MOF-74 as our model system. This MOF, first synthesized in 2005,^31^ features chains of Ni^2+^ linked together by 2,5-hydroxyterepthalate to give rise to cylindrical pores (Fig. 1a). The Ni sites typically feature 6-fold coordination but upon solvent exchange and removal can become 5-coordinated, leaving open an uncoordinated site that has previously proven to be beneficial for CO_2_ sorption (Mg, Ni, Co, Zn-MOF-74),^32^ toxic gas adsorption (Mg, Ni, Co, Zn-MOF-74),^33^ and NO_*x*_ reduction(Mn, Co-MOF-74).^34^ It is important to note that both, the carboxylate and alcohol groups of the 2,5-hydroxyterepthalate linker bind with the Ni chains. Therefore, the partial incorporation of 2-hydroxyterepthalate in place of the original linker would result in 4-coordinate Ni centers what we hypothesized to be exceptionally active in an electrochemical context (Fig. 1b–d). As a recent reference point, the coordinatively unsaturated Ni–O_4_ centers at the edges of NiOH_2_ nanoribbons were found to be highly efficient for electrocatalytic water and methanol oxidation.^35,36^ In the last year, transition metal sites in MOFs explored as active sites for HMF oxidation.^37,38^ We moved to systematically study the structure and activity of these missing hydroxyl linker defect NiO_4_ sites within Ni-MOF-74 with the aim of generating an in depth understanding of how they function in an electrocatalytic context and by doing so, extract out valuable design principles that can be generalized across a wide array of systems.

## Results and discussion

**Fig. 2:**
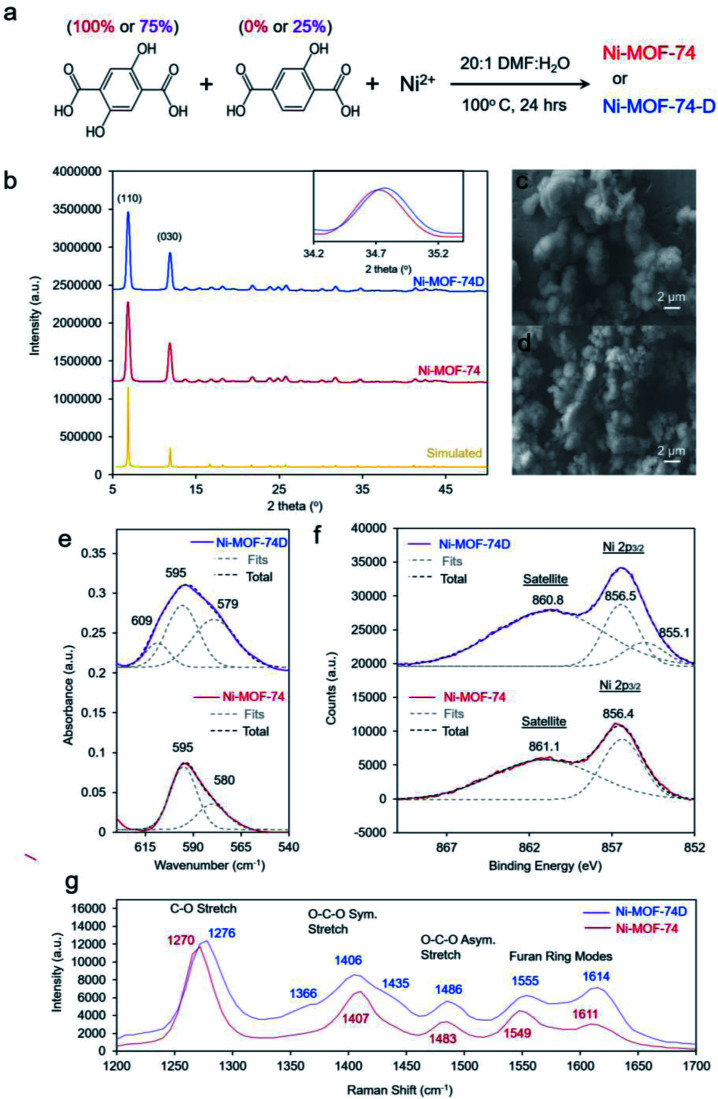
The synthesis of the Ni-MOF-74 and Ni-MOF-74D MOFs entailed the use of only dihydroxy and a combination of mono and dihydroxyterepthalic acid linkers (a). Both MOFs exhibited the same XRD spectrum, through Ni-MOF-74D featured a lower degree of crystallinity (b). Both MOFs were micron sized (c and d), though the Ni–O absorption band of Ni-MOF-74D was broader and composed of three distinct components (e). XPS spectra (f) further indicate a more electron-rich Ni electronic structure of Ni-MOF-74D and this is also reflected in the Raman spectra (g) which shows blue-shifted bands in the fingerprint region.

We proceeded to synthesize our target materials with a simple one-pot solution phase approach, adapting a previously established recipe in which missing hydroxyl linker defects were generated in Co-MOF-74.^39^ The nominally defect-free MOF, Ni-MOF-74, was synthesized with only 2,5-dihydroxyterepthalic acid while the defect-containing MOF, Ni-MOF-74D, was synthesized using a 3 : 1 ratio of 2,5-dihydroxyterepthalic acid : 2-hydroxyterepthalic acid (Fig. 2a). After synthesis, solvent exchange and evacuation, the two MOFs were first characterized with *ex situ* methods. The powder X-ray diffraction (XRD) pattern for both MOFs was well-matched to what was expected for MOF-74 (Fig. 2b). The main difference noticeable in the spectra was that Ni-MOF-74D features slightly broader peaks which were shifted to higher 2-theta values, indicating a lower degree of crystallinity and a smaller unit cell, both expected observations for defect-containing MOFs.^40–42^ Additional reflections pointing to a periodicity in the defects was not observed in the Ni-MOF-74D XRD pattern so we therefore assume the defects to be distributed without any particular ordering (Fig. S1†).

Scanning electron microscopy (SEM) revealed the two MOFs to consist of particles on the order of 2–5 micrometers in diameter without any particular shape (Fig. 2c and d). However, noticeable differences were observed in the infrared (IR) spectrum. The IR absorption bands were broader for (Fig. S2†), indicating a lower degree of homogeneity as already evidenced through XRD measurements. However, the most interesting aspect in the IR spectra was the band attributed to the Ni–O vibrations centered around 595–598 cm^−1^ (Fig. 2e). Previous studies of nickel oxides and hydroxides have recorded characteristic bands in this region.^19,43–45^ For Ni-MOF-74, the spectra could be well-fit with two peaks at 595 and 580 cm^−1^. However, the broader band of Ni-MOF-74D had to be fit with a combination of 3 peaks, at 595, 579 and 609 cm^−1^. This indicates that there is a greater distribution of coordination environments of the Ni centers within Ni-MOF-74D, as expected for a defect containing MOF.

X-ray photoelectron spectroscopy (XPS), probing the Ni 2p_3/2_ peak revealed that both MOFs featured a prominent peak at 856.4–5 eV, closely matching that of many common Ni^2+^ species such as Ni(OH)_2_ (Fig. 2f).^46^ However, Ni-MOF-74D also featured a second component centered at 855.1, attributed to the NiO_4_ defect sites. An extra band can also be seen at this position in the difference spectra (Fig. S3a†). The results therefore indicate that the Ni species within the defect sites have a higher electron density, which seems intuitive as they have less bonds electronegative oxygen species. The O 1s peak of Ni-MOF-74D is also shifted to lower binding energy relative to that of Ni-MOF-74 (Fig. S3b†). Similarly, the Raman spectra of the two MOFs illustrate that the Ni-MOF-74D bands attributed to the C–O, O–C–O, and furan ring stretches are broadened and blue-shifted (Fig. 2g), consistent with the notion that the organic linkers are bound to Ni centers with differences in electronic structure.^47,48^ This is also reflected in shifts and broadened features in the Ni–O bands in the low frequency of the Raman spectrum (Fig. S4†).

**Fig. 3:**
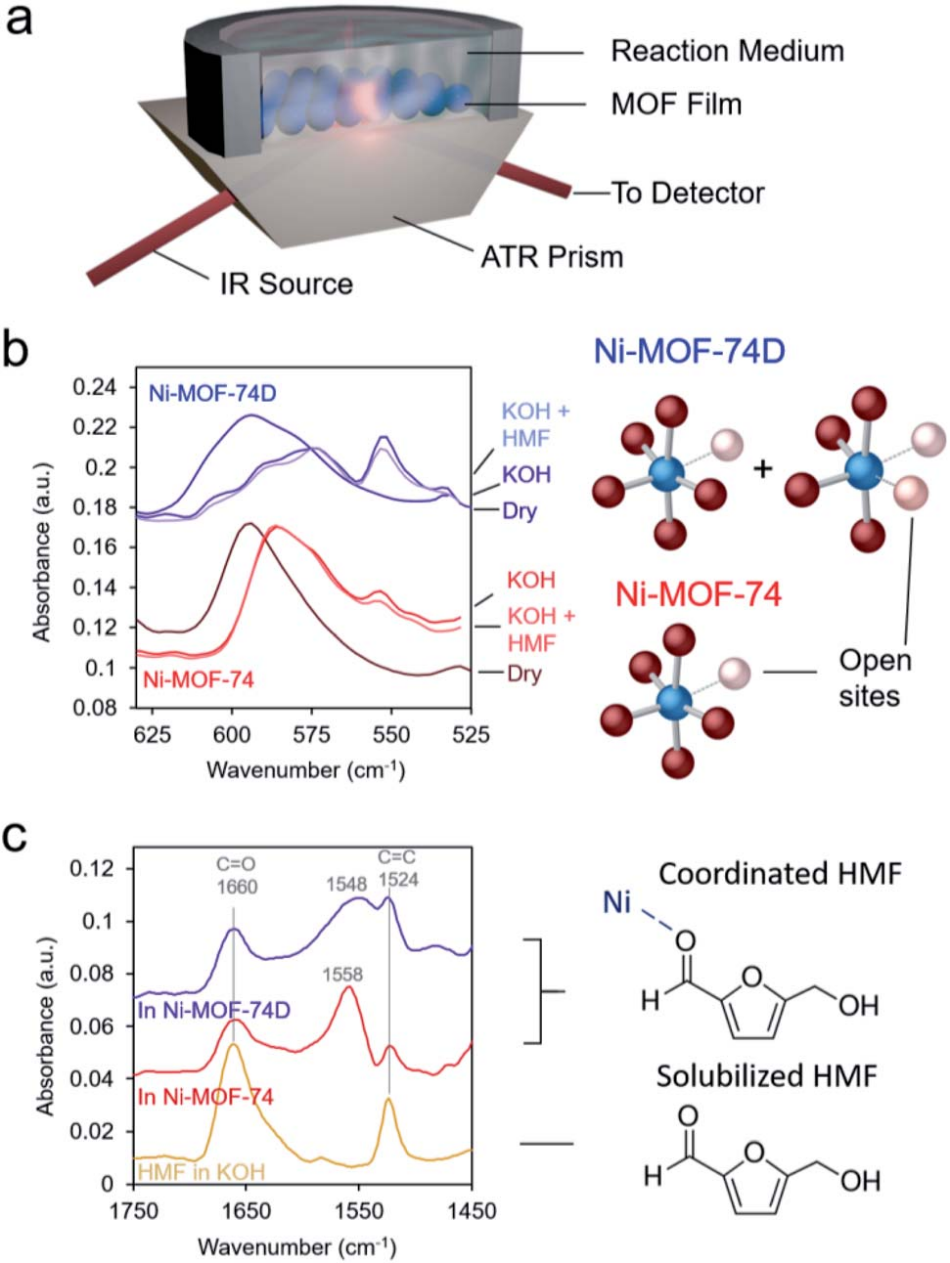
An ATR configuration was used to probe the MOF-HMF interactions (a). The low wavenumber spectra reveal that Ni-MOF-74D has more open coordination sites in which OH can adsorb, which is then partially displaced by HMF (b). The HMF-Ni interactions can also be visualized in the high wavenumber region in which a new band arises, stemming from the interaction of the HMF aldehyde group with the Ni centers of the MOFs (c).

As spectroscopic measurements can be exceptionally instrumental in understanding catalyst–reactant interactions^49^ and are increasingly used to probe MOF materials,^50^ we therefore moved to study the MOFs, and in particular their interactions with the electrolyte and HMF with IR spectroscopy in an ATR configuration (Fig. 3a). The first area of interest was the low-frequency region in which the Ni–O vibrations could be found. Upon immersion in 10 mM KOH electrolyte, the Ni–O bands red-shifted for both Ni-MOF-74 and Ni-MOF-74D (Fig. 3b), as expected due to differences in environment and presence of hydrogen bonding. Interestingly, a new peak appeared at 554 cm^−1^ that we tentatively attribute to OH interacting with the open metal sites. Bands attributed to Ni–O–H vibrations on NiOH_2_ been previously observed at this position in IR measurements.^19,44,51^ The new band was much stronger on Ni-MOF-74D as this material had more open metal sites onto which OH could adsorb.

The interaction of the HMF with the Ni centers of the two materials was subsequently probed in the high-frequency region in which the C=C and C=O stretching vibrations exist. After carefully subtracting out the spectra of the hydrated MOF, we were able to isolate the spectral features of the HMF (Fig. 3c). The bands at 1660 and 1524 cm^−1^ correspond to HMF in the solution that was not interacting with the MOF. However, a new band arose centered 1548–1558 cm^−1^ in the presence of the MOFs. The band was red-shifted and broader for Ni-MOF-74D. The broadening indicates that there is likely a distribution of different interactions present as Ni-MOF-74D features both the 4-coordinate and 5-coordinate Ni–O centers that can interact with HMF. We attribute this new band to the C=O stretch as the C=O bond of the aldehyde group is weaker upon interacting with the Ni centers and thus the band is re-shifted. Within the same line of reasoning, the redshift of the coordinated aldehyde band would also provide evidence for a stronger interaction of HMF with the Ni-MOF-74D. New emergent bands in this spectral region have been observed before for HMF interacting with Au, NiOH_2_/NiOOH and CoO_*x*_ surfaces.^52,53^ In contrast, IR and SFG studies have previously probed the process of HMF oxidation on Ni,^54^ nickel boride^55^ and nickel nitride^56^ catalysts, respectively, but only the formation solution-based species like 5-hydroxymethyl-2-furancarboxylic acid were detected rather than surface-interacting species. The displacement of water of hydroxyl ligands by HMF is also evidenced through potentiometric titration, in which the open circuit potential of the Ni-MOF-74D changes with increasing HMF addition to the electrolyte (Fig. S5†).

**Fig. 4:**
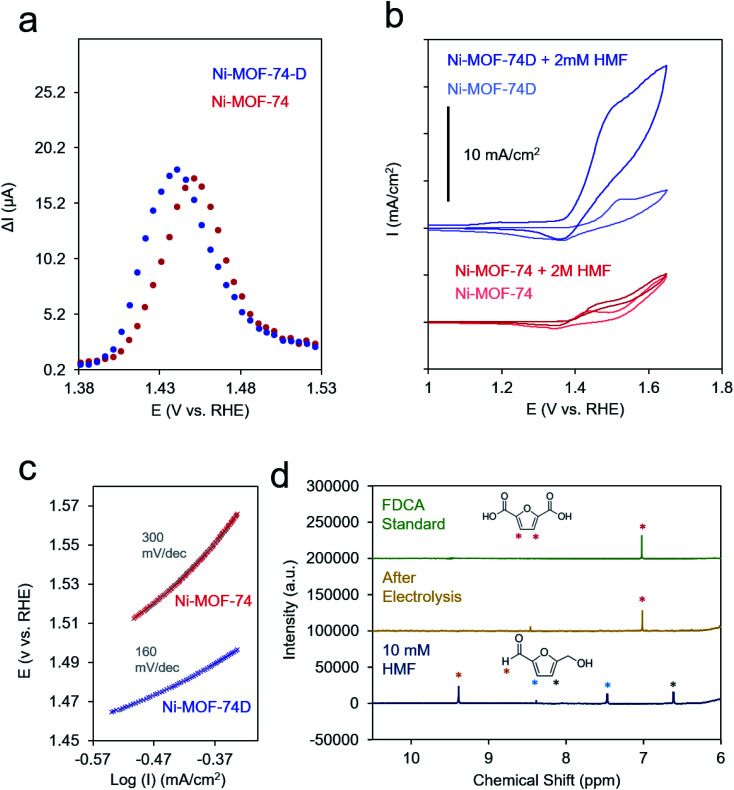
DPV measurements illustrate differences in the Ni redox potentials of Ni-MOF-74D and Ni-MOF-74 (a) while CV measurements in the presence of 2 mM HMF highlight the enhanced catalytic activity of Ni-MOF-74D (b). Catalytic differences are also evident by differences in Tafel slopes in a rotating disk configuration (c). HMF could be completely converted to FDCA after extended electrolysis as shown in NMR spectra (d).

We finally set out to study the catalytic effect that the defect sites imparted onto Ni-MOF-74D with an array of electrochemical measurements in a 10 mM KOH electrolyte. Briefly, electrodes were constructed by making a catalyst ink by sonicating together carbon nanotubes, Nafion, and the desired material in a 3 : 1 vol:vol ethanol : water solution (10 mg ml^−1^). This ink was drop cast onto a carbon paper substrate to attain a final MOF loading of 1 mg cm^−2^ and allowed to dry in ambient conditions. Because of the relatively low conductivity of both MOFs (Fig. S6†), differential-pulse voltammetry (DPV) was used to prove the Ni(ii/iii) redox potential. The redox wave of Ni-MOF-74D was broadened and shifted to more negative potentials relative to Ni-MOF-74, indicating that both, a distribution of redox environments existed (*e.g.* 4- and 5-coordinated Ni–O) and that the 4-coordinate Ni centers overall had a more negative redox potential (Fig. 4a).

In the cyclic voltammetry (CV) measurements at 20 mV s^−1^, Ni-MOF-74 featured only a modest increase of current between 0.9 and 1.7 V upon the addition of 2 mM HMF to the electrolyte solution (Fig. 4b). In contrast, Ni-MOF-74D exhibited rapidly increasing catalytic current for HMF oxidation beginning at 1.4 V. Differences in the catalytic activity of the two systems were also visible in the different Tafel slopes for HMF oxidation (160 mV dec^−1^ for Ni-MOF-74D, 300 mV dec^−1^ for Ni-MOF-74) (Fig. 4c and S6†). These measurements were taken near the onset of the catalytic current, just past the Ni(ii/iii) redox potential in a rotating disk electrode setup at slow scan rates (0.5 mV s^−1^) before mass transport limitations came into effect. After a long-term 24 h electrolysis of 10 mM HMF, the reactant could be completely converted to 2,5-furandicarboxylic acid (FDCA) at 95% yield as both the aldehyde and alcohol groups of HMF were completely oxidized to the carboxylic acid (Fig. 4e). FDCA is the highest-value product from HMF oxidation and is an important precursor to polymers. NMR spectra taken at short measurement times showed the presence of furan-2,5-dicarbaldehyde (DFF) and 5-formyl-2-furancarboxylic acid (FFCA), indicating that the reaction preferentially proceeds through the oxidation of the alcohol group of the HMF first, rather than the aldehyde group (Fig. S8†). The turnover number, determined by dividing the molecules of FDCA detected by the quantity of electrochemically addressable Ni sites, was determined to be 1520 and the faradaic efficiency for FDCA formation to be 80%, with water oxidation attributed to the rest of the charge passed. This performance is exceptional, especially in pH 12. In comparison, Au and Ni electrodes only showed minimal (<1% conversion) FDCA production under the same reaction conditions.^52,53^ MOFs and COFs have begun to be used for this reaction but have thus far only been applied in highly alkaline conditions.^37,57^ Similarly, high conversion rates and faradaic efficiency values (both above 95%) have been noted for transition metal based catalysts, but only in highly alkaline conditions (0.1 to 1.0 M KOH).^54–56,58–64^ As discussed below, we believe that the key to the high performance of this system in weakly basic conditions lies in the unique active site configuration.

At potentials more positive of the Ni(ii/iii) redox potential, products could not be reaction detected, indicating that the Ni(iii) species is necessary for sustained catalysis. The stability of the system was evaluated with a combination of SEM, XRD and Raman measurements. SEM images showed that the morphology of the Ni-MOF-74D is visibly similar after a test 24 h catalytic run at 1.4 V (Fig. S0†). Similarly, the expected diffraction pattern could still be detected after catalysis (Fig. S10a†) and the Raman spectrum retains all of the features of the Ni–O bands and the linker vibrations (Fig. S10b†).

**Fig. 5:**
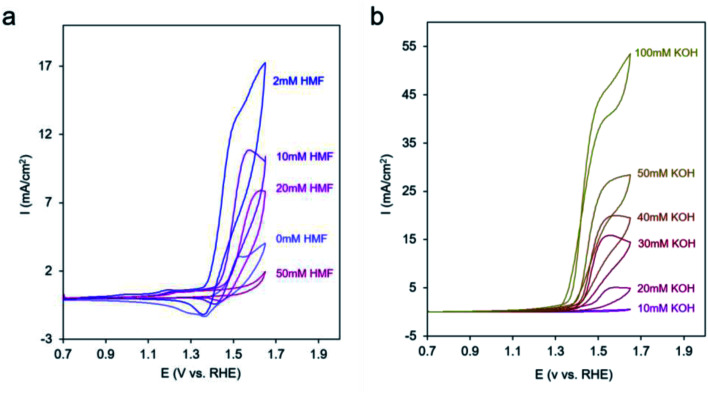
CV cycles with Ni-MOF-74D under 10 mM KOH upon increasing the HMF concentration (a) and at 50 mM HMF with increasing the KOH concentration (b).

In an effort to probe the precise mechanism occurring within Ni-MOF-74D, we first moved to vary the concentration of HMF in the solution. Surprisingly, we saw a decrease in catalytic current as the HMF concentration increased (Fig. 5a) to the point that 50 mM HMF almost completely suppressed the current. However, there was still an increase of current at potentials below 1.3 V attributed to the HMF adsorption process onto the Ni sites (Fig. S11†). In contrast, we did not observe such a phenomenon on Ni-MOF-74, indicating that the defect Ni sites are more active for this adsorption process (Fig. S12†). The suppression of catalytic activity points to a surface poisoning of the Ni sites when excessive HMF is present and points the notion that the HMF is not the only participant in the reaction process. Initial speculation at this stage pointed to co-adsorbed *OH from the electrolyte as necessary. Previous work on heterogeneous alcohol oxidation catalysts demonstrated that *OH not only stabilizes reaction intermediates on heterogeneous catalyst surfaces in the alcohol oxidation process through hydrogen bonding but also functions as a proton acceptor and/or becomes incorporated into the final product.^65–68^ In our system, *OH is the first intermediate of the oxygen evolution reaction and can only form if the Ni-MOF-74D surface is not completely saturated with HMF.

After diminishing the catalytic activity with 50 mM HMF, we found that the activity can be recovered and even surpassed when KOH was systematically added to the electrolyte (Fig. 5b). This may be due to either OH^−^ in solution now playing the role of proton acceptor or to a competitive binding between HMF and OH^−^ that once more enables the aforementioned reaction pathway. In contrast, when KOH was added to a solution with only 2 mM HMF, only modest enhancements of reaction rates were observed (Fig. S13†).

**Fig. 6:**
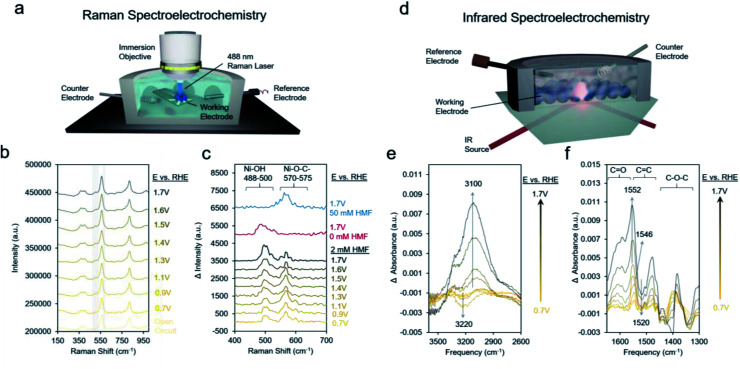
Raman setup used for spectroelectrochemical measurements (a). Absolute (b) and difference spectra (c) illustrate that both *OH and adsorbed HMF are necessary for the reaction mechanism in 10 mM KOH conditions. IR spectroscopy under the same conditions with the setup illustrated in (d) shows the adsorption of *OH (e) and changes in surface bound HMF as it is oxidized to DFF (f).

We proceeded to use Raman spectroscopy with a home-built *in situ* reaction cell integrated with a water immersion objective (Fig. 6a) to see if we can provide evidence for our hypothesis that neighboring *OH species are necessary for HMF oxidation. Raman spectra on Ni-MOF-74D with 10 mM KOH and 2 mM HMF from open circuit (around 0.5 V) to 1.7 V showed two new bands arising around 495 cm^−1^ and 575 cm^−1^ (Fig. 6b). NiOOH, typically considered to be the active phase of Ni-based oxides for the oxygen evolution reaction and HMF oxidation reactions features strong Raman bands around 475 and 555 cm^−1^ was not observed here pointing to a lack of restructuration of the Ni-MOF-74D active sites.^69^ Further, the addition spectral features, which are more evident in the difference spectra (Fig. 6c), form prior to the redox feature observed in the Ni-MOF-74D CV. However, the Ni–OH band of nickel hydroxide is typically found at 494 cm^−1^.^70^ Thus, tentatively attribute this band to the Ni–O vibrational mode of *OH adsorbed onto a Ni site of Ni-MOF-74D. This band also appears when HMF is not present in solution supporting this assignment. The band around 570–575 cm^−1^ is, on the other hand, likely a Ni–O stretch of an HMF bound *via* the alcohol's oxygen atom onto the Ni-MOF-74. This band is visible in 50 mM HMF as well, but not the lower-frequency band around 488–500 cm^−1^. This strengthens our previous statement that the saturation of available Ni sites by HMF occurs in 50 mM HMF and acts as a poison, preventing the co-adsorption of OH^−^ species that participate in the reaction process. As the reaction proceeds, first the adsorbed HMF band is dominant, and at potentials more positive of the Ni(ii/iii) redox potential, the HMF band diminished and the *OH band becomes dominant, reflective of the changes in surface-adsorbed species once the catalytic cycle initiates.

Likewise, infrared spectroelectrochemistry (simplified setup shown in Fig. 6d) was used as a complementary probe of changes in the surface reaction intermediates on the Ni sites of Ni-MOF-74D. With the Ni-MOF-74D MOF at open circuit serving as the reference and as the potential was gradually increased from 0.7 to 1.3 V, a negative band at 3200 cm^−1^ appeared, tentatively attributed as the O–H stretch of water molecules which were interacting with the Ni sites and began to be displaced by HMF molecules (Fig. 6e). This matches the frequency of the OH band of solvent water molecules interacting with Ni-MOF-74D in open circuit conditions (Fig. S16†). Further, OH bands of water molecules interacting with Mg-, Co- and Ni-MOF74 have been observed in this region and the red-shift relative to free H_2_O in the electrolyte suggests the presence of a strong hydrogen bonding network.^71–73^ As the potential was increased further to 1.7 V, a positive band arose at 3100 cm^−1^, here attributed to *OH adsorbing onto Ni sites as part of the OER cycle. In the same potential range, first a band at 1546 cm^−1^ appeared at potentials prior to the catalytic onset, having a position matching that of the intermediate band from the open circuit measurements in Fig. 3c. Once more positive potentials were applied and catalysis initiated, a positive band at 1552 cm^−1^ arose and a downward pointing feature at 1520 cm^−1^ also appeared. Only HMF (amongst HMF, DFF, HMFCA, FFCA and FDCA) has a strong band at 1520 cm^−1^ and this is attributed to the conversion of HMF. Changes around 1025 cm^−1^ corresponding to the HMF alcohol group are also visible (Fig. S17†), matching closely to HMF conversion to DFF formation as the first reaction step.

**Fig. 7:**
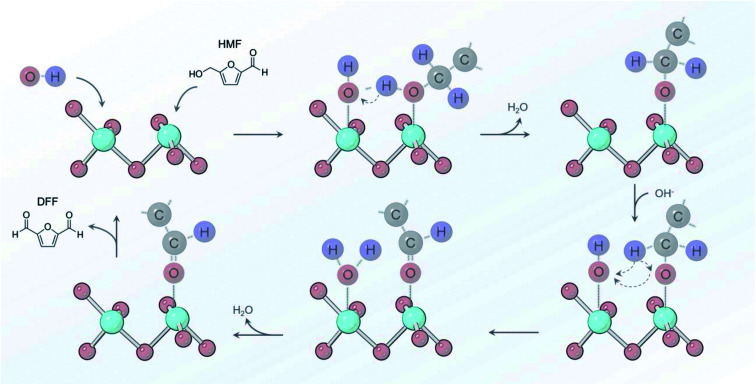
Reaction pathway proposed for oxidizing the alcohol group of HMF that involves *OH as a key proton acceptor in pH 12 electrolyte.

From the sum of the spectroscopic and electrochemical data, there are several factors that we can put forth a reaction mechanism that occurs on Ni-MOF-74D in 10 mM KOH conditions (Fig. 7). An initial adsorption of both OH^−^ and HMF is necessary and only occurs in relatively low HMF concentrations (below 50 mM) as HMF would otherwise saturate the surface. *OH water oxidation intermediates have been shown to facilitate alcohol oxidation reactions and are crucial here.^65,74–76^ *OH accepts the proton from the alcohol group and the adjacent carbon and leaves as H_2_O back into solution. Upon oxidizing the aldehyde groups, *OH is added to the surface intermediate to eventually for the carboxylate group. In both of these cases, Ni-MOF-74D is unique in that its high density of undercoordinated active sites facilitate this pathway as Ni catalysts feature only minimal activity in pH 12.^53^ HMF adsorption occurs prior to the Ni(ii/iii) redox potential but catalysis only initiates once the Ni(iii) oxidation state is attained.

## Concluding remarks

In summary, we present a combined spectroscopic and electroanalytical study on the catalytic properties of defect sites in Ni-MOF-74. Using electrochemical HMF oxidation as a model reaction, we show through *in situ* vibrational spectroscopy and electrochemical analysis that there are distinct Ni coordination environments in the defect containing MOF that consequently lead to unique interactions with HMF and therefore exceptional electrocatalytic performance. By having precisely defined active sites, we extract a reaction mechanism that involves surface-bound *OH that participate in the reaction mechanism by accepting protons from HMF intermediates en route to DFF and eventually FDCA. We note that extending this strategy to other MOF systems can be readily carried out, as with Co-MOF-74D (Fig. S18 and S19†). Most importantly, the conceptual knowledge developed is not limited to HMF oxidation but can be extended to a variety of emerging electrosynthetic processes gaining importance in the context of renewable energy research.

## Author contributions

All authors contributed to the design and execution of the experiments and to generation of the manuscript.

## Conflicts of interest

None to declare.

## Supplementary Material

SC-012-D1SC00573A-s001
