# Supplementary material for: Rational incorporation of defects within metal–organic frameworks generates highly active electrocatalytic sites
Source: Chem Sci. 2021 Apr 29;12(21):7324–33. doi: 10.1039/d1sc00573a (PMC8171315; doi:10.1039/d1sc00573a)
Supplement: SC-012-D1SC00573A-s001 [file SC-012-D1SC00573A-s001.pdf]

Electronic Supplementary Material (ESI) for Chemical Science.
This journal is © The Royal Society of Chemistry 2021

Supporting Information for:

Rational incorporation of defects within metal-organic frameworks generates highly active electrocatalytic sites

Nina Heidary[1], Daniel Chartrand[1], Amandine Guiet[2], and Nikolay Kornienko[1]*

[1]Department of Chemistry, Université de Montréal, 1375 Avenue Thérèse-Lavoie-Roux, Montréal, QC H2V 0B3, Canada.

[2]Institut des Molécules et Matériaux du Mans (IMMM), UMR 6283 CNRS, Le Mans Université, Avenue Olivier Messiaen, 72085 Le Mans

*E-mail: nikolay.kornienko@umontreal.ca

MOF Synthesis: The MOFs were synthesized in a one-pot solution phase method. 20 mL scintillation vials were filled with 10 mL of DMF, 0.5 mL of water, 136.5 mg of nickel nitrate hexahydrate, and 52 mg of 2,5-dihydroxyterepthalic acid. For Ni-MOF-74D, 39 mg 2,5-dihydroxyterepthalic acid and 13 mg 2-hydroxyterepthalic acid were used. The solutions were briefly sonicated to dissolve all of the components and placed into an oven at 100°C for 24 hrs. Following the MOF growth, the residual solvent was removed by soaking in methanol at 70°C for 5 days while periodically refreshing the methanol and finally vacuum drying at 80°C for 3 days.

Physical Characterization: XRD spectra were taken on the MOF powders both after synthesis and after electrochemical measurements in which the MOF electrodes were simply rinsed with water and allowed to dry in an air environment. For SEM measurements, the MOFs were dispersed in ethanol and drop cast onto titanium foil or the MOF electrodes (described below) were imaged after simply rinsing with water.

Infrared Spectroscopy:

A ThermoFischer Nicolet 380 FTIR-ATR was used for all IR measurements. The MOFs, either dry or dispersed in KOH or HMF were pressed directly onto a diamond coated ZnSe ATR waveguide. An atmospheric background spectrum was taken before every MOF spectrum. Each spectrum was recorded with an accumulation of 400 scans with a resolution of 4 cm^{-1}. OPUS 7.0 software was used to carefully subtract out the air and KOH background spectra. This software was also used for baseline correction and peak fitting.

Electrochemistry: For all electrochemical experiments a Biologic VMP 200 potentiostat, coupled with EC-lab software was utilized. Custom-built 2-compartment glass electrochemical cells were used. Ag/AgCl reference electrodes and graphite rod counter electrodes were employed in electrochemical measurements. Periodically, the Ag/AgCl reference electrode was checked vs. a master electrode to ensure that the potential did not significantly drift and affect the measurements. 10mM KOH with 1M KCl supporting electrolyte was used for all measurements. The solution resistance was compensated for at 85% with the ZIR function in EC-Lab, which measures this value at open circuit potentials at 100 KHz frequency.

To fabricate working electrodes, first a catalyst ink was made from 150 µL water, 450 µL ethanol, 5 µL nafion solution (5% wt. in water), 6 mg MOF, and approx. 0.1 mg multiwall carbon nanotubes (40 nm diameter). This solution was sonicated for 20 minutes to render it homogeneously dispersed and was subsequently drop cast onto either toray carbon paper or onto the glassy carbon rotating disk electrode surface and let dry in ambient conditions for 20 minutes prior to use. The use of the this particular formulation was to minimize the ohmic losses through adding a conductive binder (carbon nanotubes) and to minimize the MOF detachment from the electrode via adding in the nafion component. Product quantification was performed with NMR measurements with an acetate internal standard and calibration curves performed with known concentrations of standards. In extended bulk electrolysis runs, the operating current density was approximately 2 mA/cm^2.

Raman Spectroscopy: Raman spectroscopy was conducted using a Renishaw Invia system and a 488 nm laser in all experiments. A line-focus illumination technique spread out the laser beam over a large area without minimizing overall power to minimize the laser intensity on any one point of the system. A custom-built Teflon in situ Raman cell was used, using a carbon counter electrode and Ag/AgCl reference

electrode (WPI instruments, Dri-ref). The same carbon paper/Ni-MOF-74D catalyst was used as for the conventional electrochemical experiments. All measurements were conducted with a Biologic VMP200 potensiostat. Typical accumulation times were 200s per spectrum. Throughout a chronoaperometric potential step measurement, the same spot on the catalyst was measured.

X-ray Photoelectron Spectroscopy: A VG Escalab 220i-XL instrument with a Mg emission source at the Institute National de la Recherche Scientifique Centre Énergie Matériaux et communications was employed for XPS measurements. To calibrate the energy scale, the carbon 1s peak was used as an internal standard and set to 284.8 eV. Prior to fitting, the background was subtracted with a Shirley-type background function.

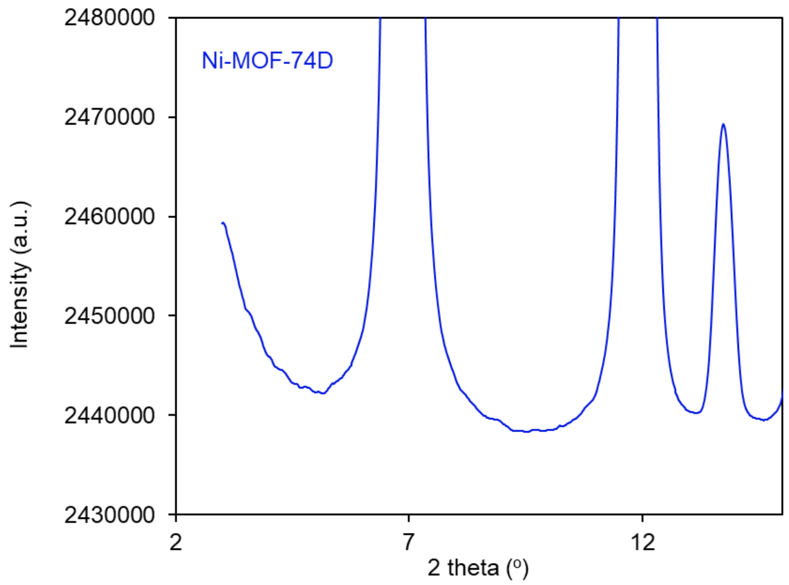

Figure S1: XRD spectrum of Ni-MOF-74D zoomed in shows a lack of additional peaks at low wavenumbers and therefore no evidence for ordering of the defects

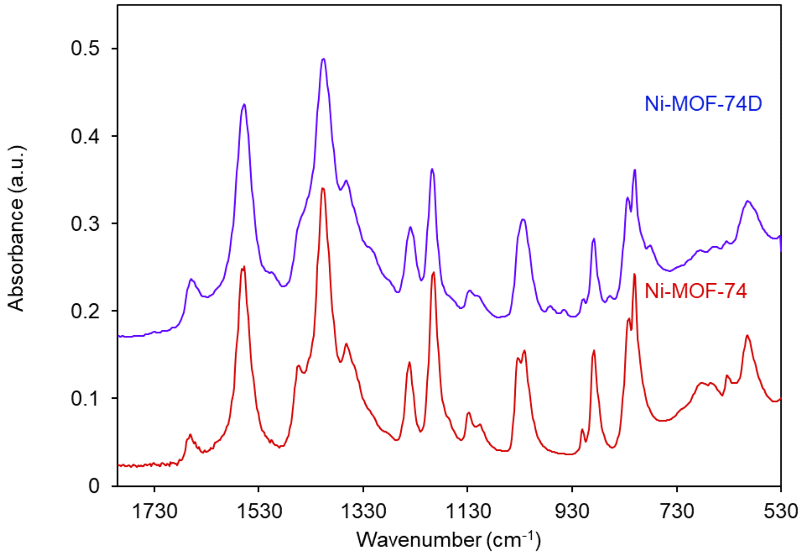

Figure S2: IR spectra of dry Ni-MOF-74 and Ni-MOF-74D MOFs show a peak broadening of Ni-MOF-74D, consistent with a decrease in crystallinity.

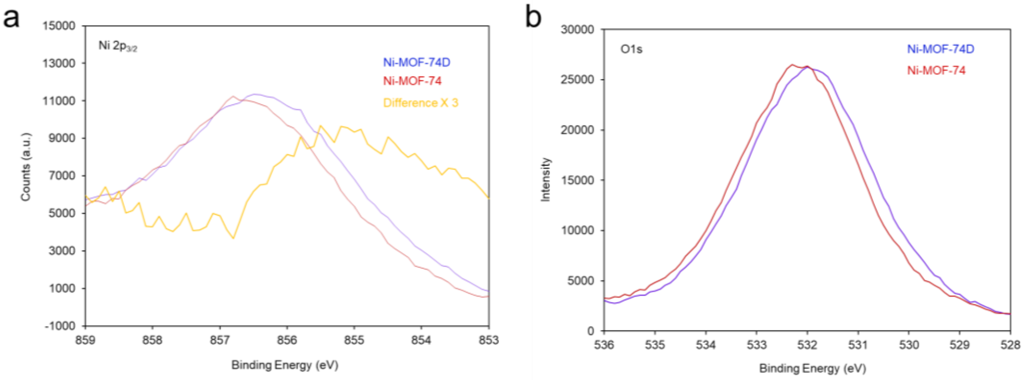

Figure S3: Zoomed-in XPS spectra of Ni-MOF-74 and Ni-MOF-74D highlighting differences for the $Ni2p_{3/2}$ peaks (a) and O1s peaks (b).

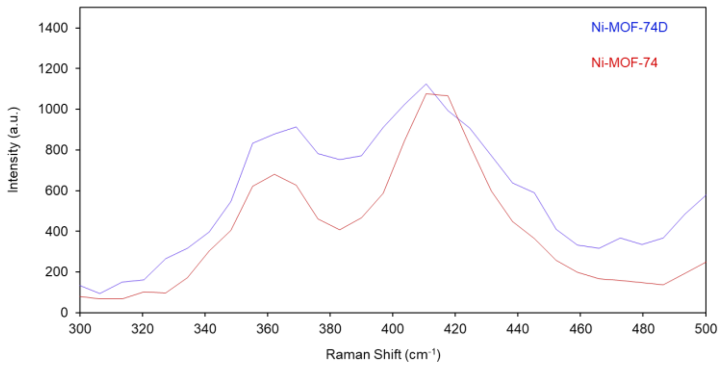

Figure S4: The low-frequency region of the Raman spectra reveals differences in the Ni-O bands of Ni-MOF-74 and Ni-MOF-74D through shifted and broadened peaks.

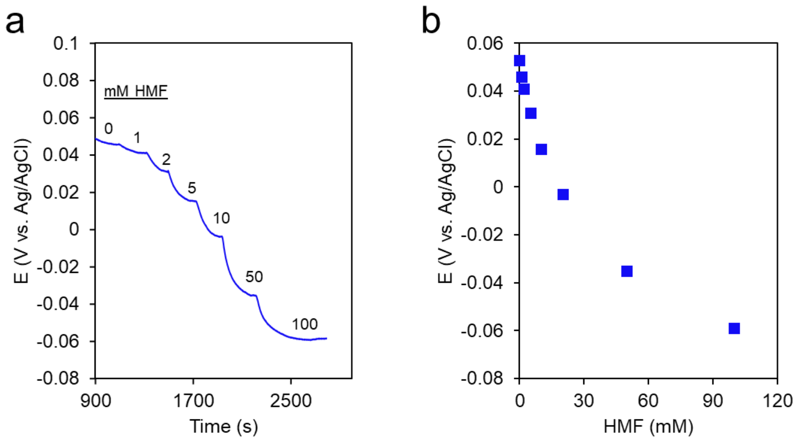

Figure S5: The open circuit potential of the Ni-MOF-74D electrode systematically changes with increasing HMF concentrations in the electrolyte (a, b). This indicates that HMF continually binds to the electrode, displacing water or hydroxyl ligands.

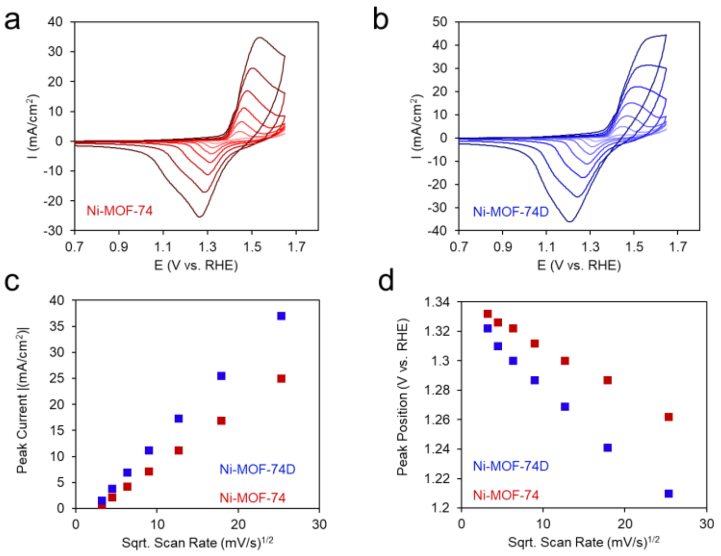

Figure S6: CVs of Ni-MOF-74 (a) and Ni-MOF-74D (b) taken at different scan rates. The peak current scales with the square root of the scan rate, indicative of diffusion-limited charge transport (c). The peak position similarly shifts with scan rate (d).

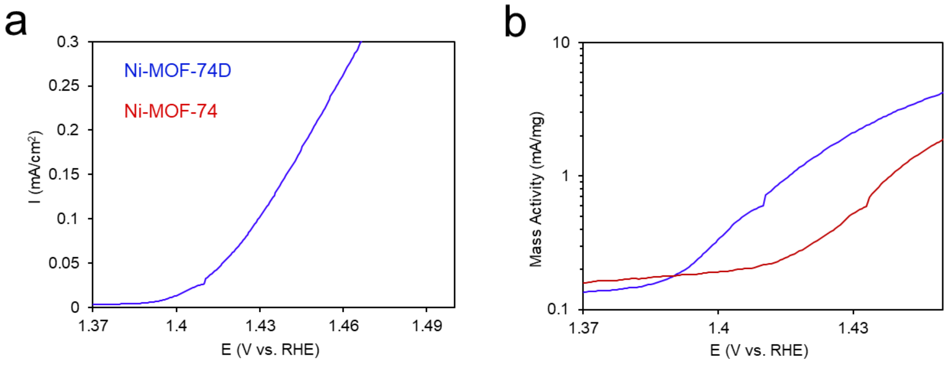

Figure S7: Linear sweep voltammograms (a) and mass activity (b) of Ni-MOF-74 and Ni-MOF-74D with 2mM HMF at 0.5mV/s scan rate in a RDE setup (1600 RPM).

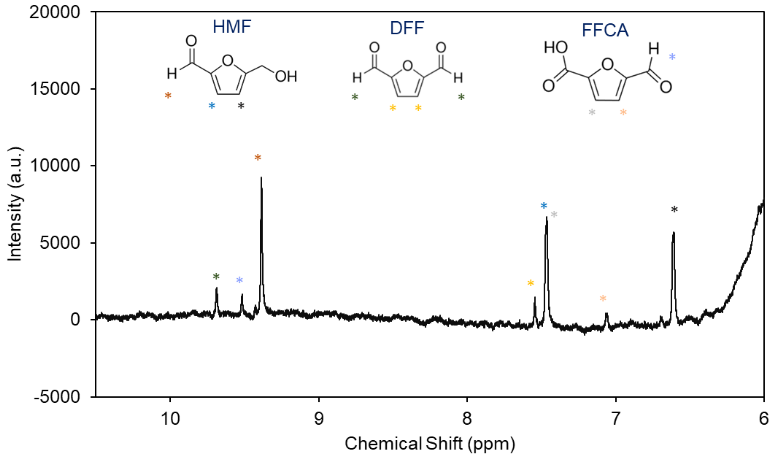

Figure S8: NMR spectra of HMF oxidation of Ni-MOF-74D taken at 2 hrs reveals the formation of DFF and FFCA.

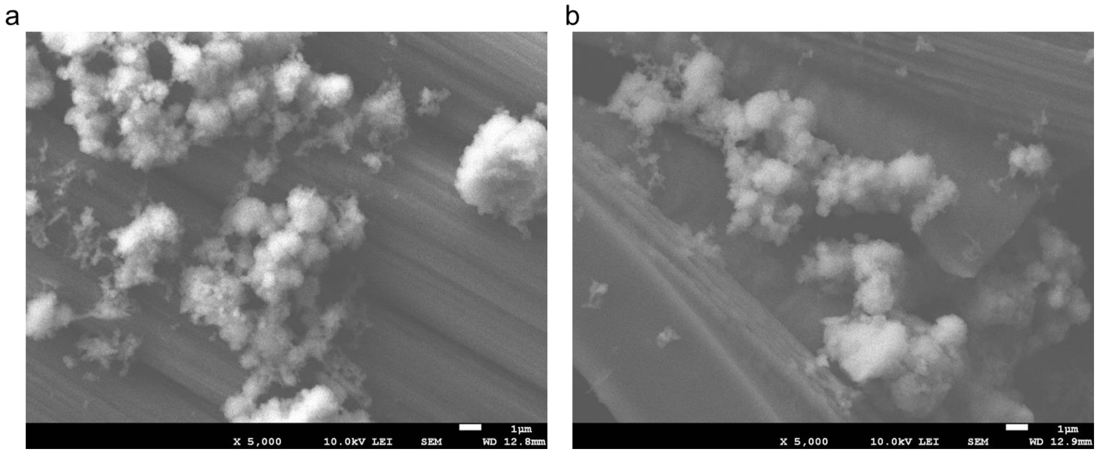

Figure S9: Representative SEM images of Ni-MOF-74D before (a) and after (b) electrolysis for 24 hrs.

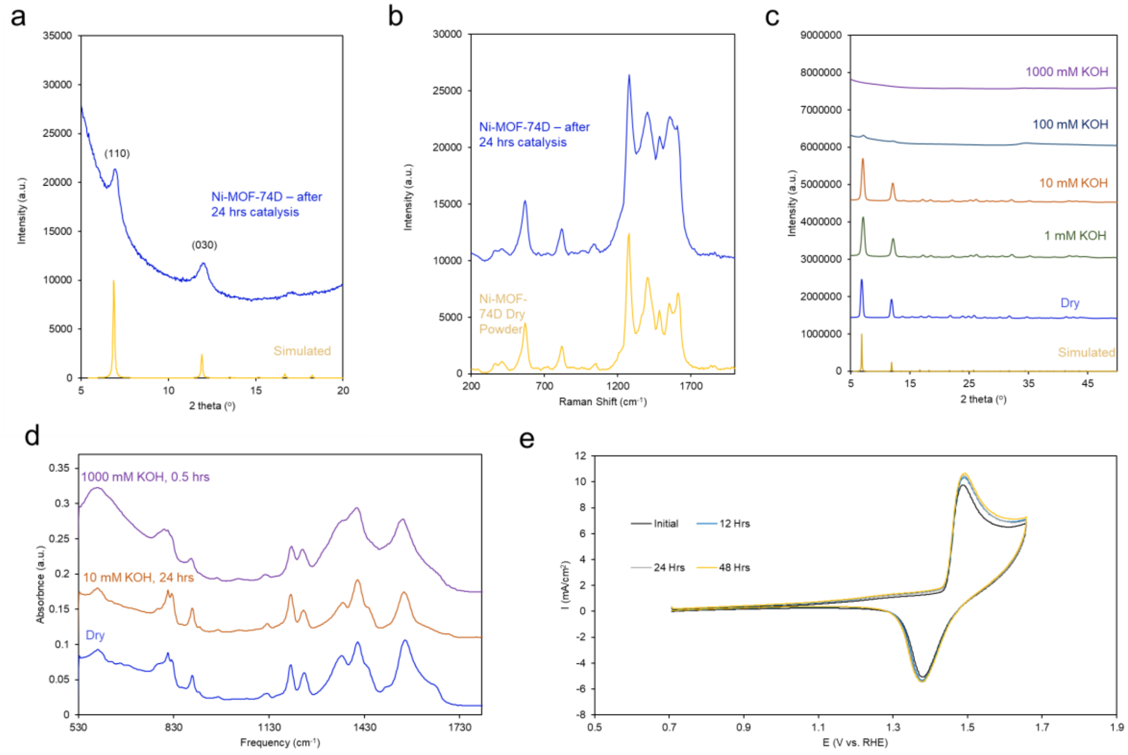

Figure S10: PXRD pattern of Ni-MOF-74D after 24 hrs of electrolysis show that the principal (110) and (030) peaks remain (a) and the Raman spectra similarly show the retention of the Ni-O bands as well as the linker vibrational modes (b). The PXRD patterns of the MOF powders soaked in varying KOH concentrations shows that a loss of crystallinity occurs only in solutions of 100 mM and 1000 mM KOH (c). This is also evident by the rapid broadening of the IR bands after only 30 min in 1000 mM KOH (d). The Ni redox signal also remain constant for up to 48 hrs of electrolysis (at 1.5V vs. RHE), indicating the retention of the Ni sites on the electrode and the retention of the Ni local environment (e).

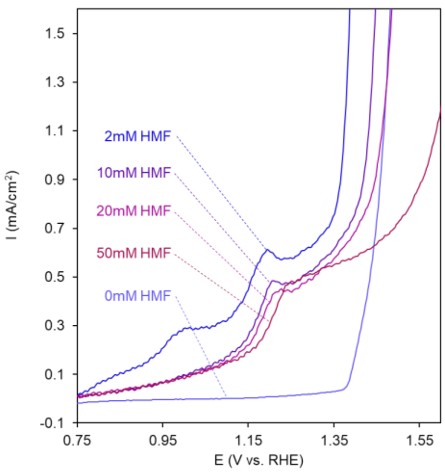

Figure S11: Zoomed in region of Ni-MOF-74D at low potentials showing the pre-catalytic HMF adsorption current.

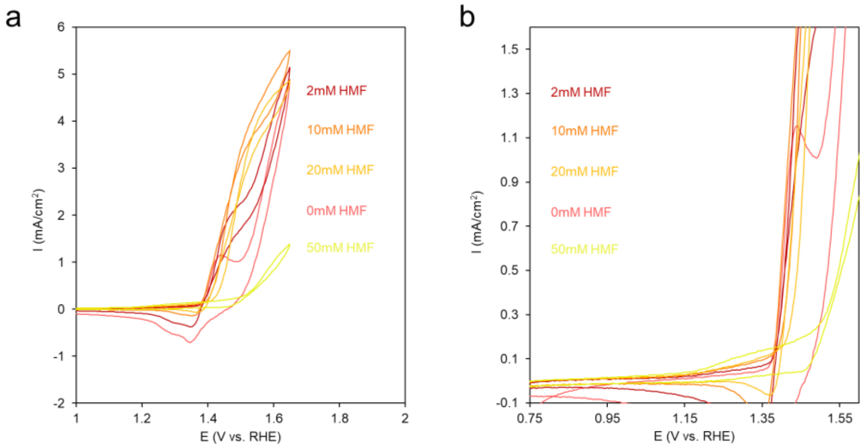

Figure S12: CVs of Ni-MOF-74 at full scale (a) and zoomed in to the pre-catalytic region (b) with different amount of HMF in the electrolyte solution.

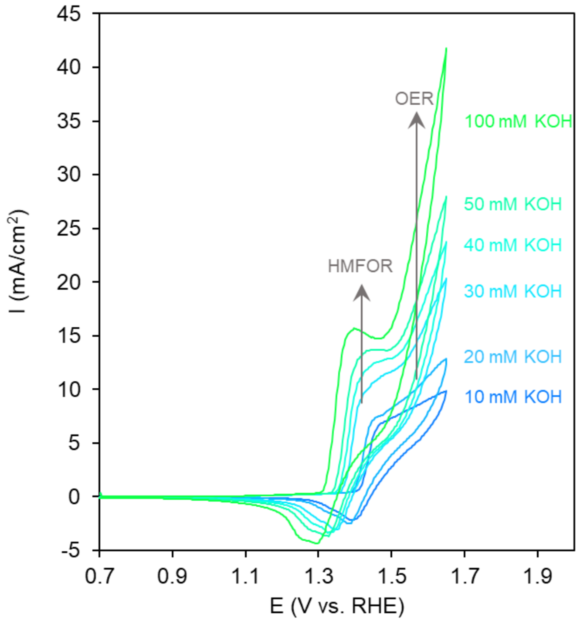

Figure S13: CVs of Ni-MOF-74D with 2mM HMF as KOH is progressively added to the electrolyte solution.

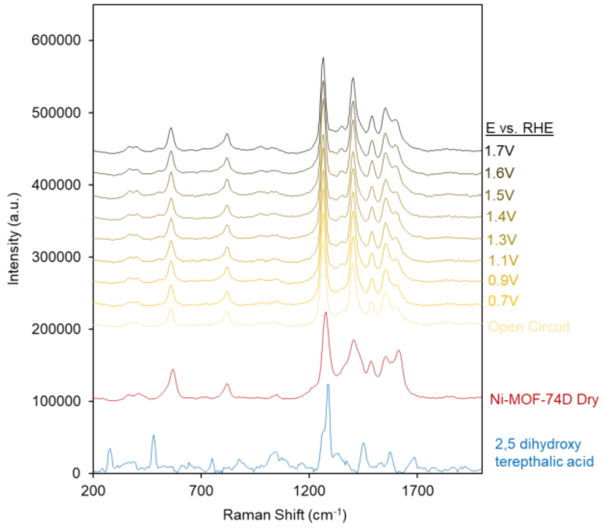

Figure S14: Raman spectra of Ni-MOF-74D, 2,5 hydroxyterepthalic acid and Ni-MOF-74D in pH12 with 2mM HMF in solution as a function of applied bias.

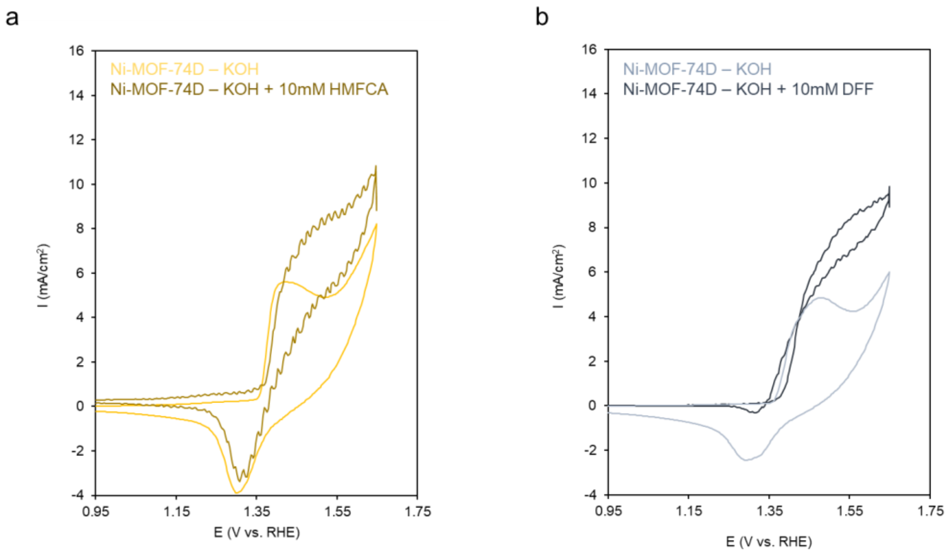

Figure S15: CVs of HMFCA oxidation (a) and DFF oxidation (b) with Ni-MOF-74D.

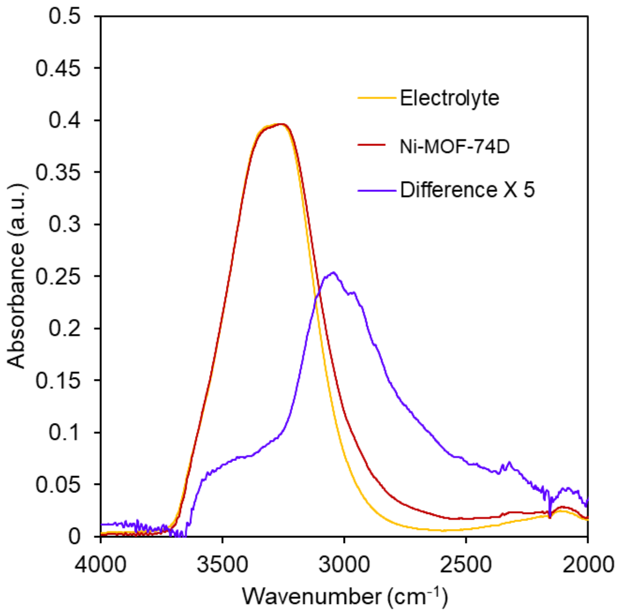

Figure S16: IR spectra of the KOH electrolyte and the electrolyte Ni-MOF-74D shows an additional band attributed to solvent interacting with the MOF.

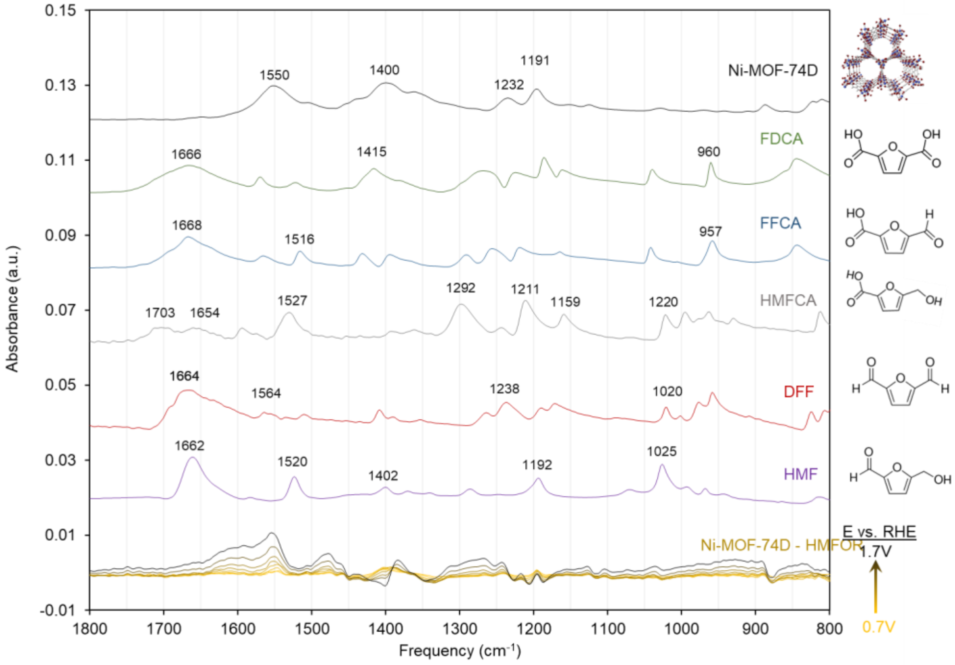

Figure S17: Reference spectra of IR spectroelectrochemistry of Ni-MOF-74D alongside those of the reactant, possible products and the spectrum of the Ni-MOF-74D MOF. All references are taken in the same KOH solution as the (spectro)electrochemical experiments are performed in.

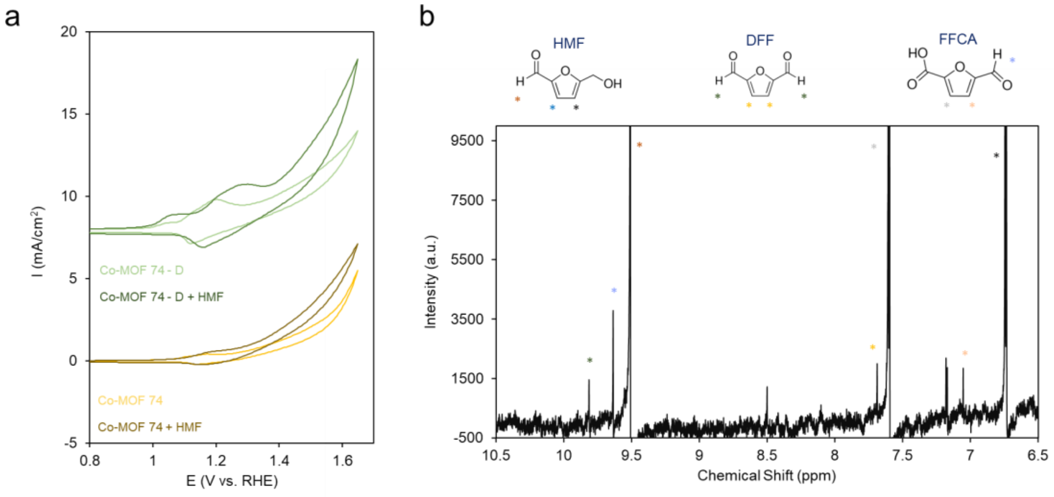

Figure S18: Electrochemical performance of Ni-MOF-74 and Ni-MOF-74D in the absence and presence of 10 mM HMF (a). Product quantification after 1h indicates that the reaction proceeds through DFF as a primary intermediate en route to FDCA formation (b).

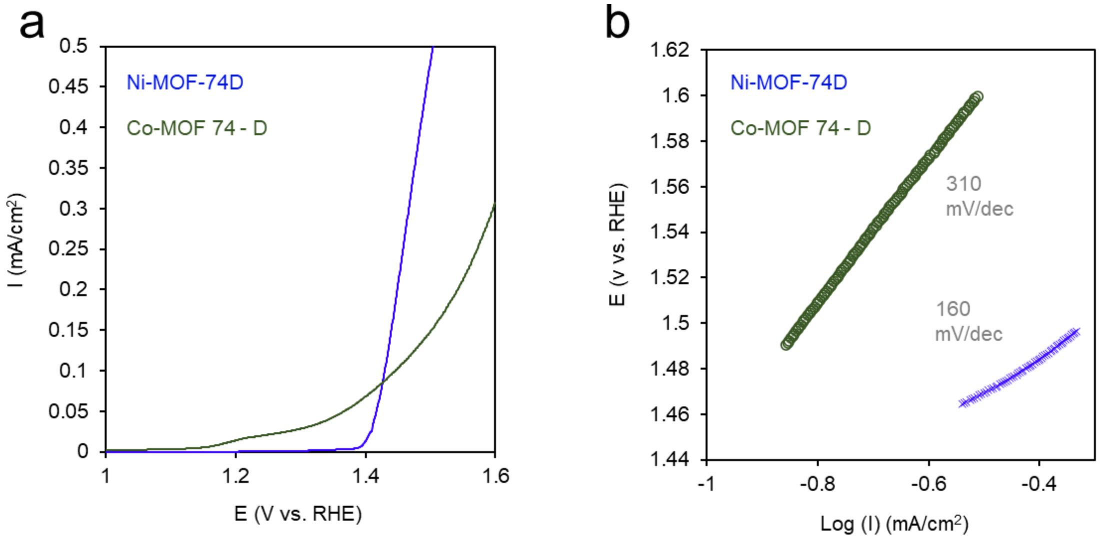

Figure S19: Linear sweep voltammograms (LSV) (a) and Tafel slope plots (b) in a RDE setup (1600 RPM, pH 12, 2mM HMF), indicate that Co-MOF-74D has an earlier onset potential but slower reaction kinetics relative to Ni-MOF-74D.s
